# Differences in State Traumatic Brain Injury–Related Deaths, by Principal Mechanism of Injury, Intent, and Percentage of Population Living in Rural Areas — United States, 2016–2018

**DOI:** 10.15585/mmwr.mm7041a3

**Published:** 2021-10-15

**Authors:** Jill Daugherty, Hong Zhou, Kelly Sarmiento, Dana Waltzman

**Affiliations:** 1 Division of Injury Prevention, National Center for Injury Prevention and Control, CDC.

Traumatic brain injuries (TBIs) have contributed to approximately one million deaths in the United States over the last 2 decades (*1*). CDC analyzed National Vital Statistics System (NVSS) mortality data for a 3-year period (2016–2018) to examine numbers and rates of TBI-related deaths, the percentage difference between each state’s rate and the overall U.S. TBI-related death rate, leading causes of TBI, and the association between TBI and a state’s level of rurality. During 2016–2018, a total of 181,227 TBI-related deaths (17.3 per 100,000 population per year) occurred in the United States. The percentage difference between state TBI-related death rates and the overall U.S. rate during this period ranged from 46.2% below to 101.2% above the overall rate. By state, the lowest rate was in New Jersey (9.3 per 100,000 population per year); the states with the highest rates were Alaska (34.8), Wyoming (32.6), and Montana (29.5). States in the South and those with a higher proportion of residents living in rural areas had higher rates, whereas states in the Northeast and those with a lower proportion of residents living in rural areas had lower TBI-related death rates. In 43 states, suicide was the leading cause of TBI-related deaths; in other states, unintentional falls or unintentional motor vehicle crashes were responsible for the highest numbers and rates of TBI-related deaths. Consistent with previous studies (*2*), differences in TBI incidence and outcomes were observed across U.S. states; therefore, states can use these findings to develop and implement evidence-based prevention strategies, based on their leading causes of TBI-related deaths. Expanding evidence-based prevention strategies that address TBI-related deaths is warranted, especially among states with high rates due to suicide, unintentional falls, and motor vehicle crashes.

NVSS collects data on all deaths that occur among U.S. residents. For this study, data from NVSS’s multiple cause-of-death files for 2016–2018 were combined to estimate the incidence of TBI-related deaths. An established surveillance definition was used to classify TBI-related deaths with codes from the *International Classification of Diseases, Tenth Revision* (ICD-10)* if 1) the single underlying cause of death was listed as an injury, consistent with previous reports (*1*), and 2) any of the multiple codes for causes of deaths listed in the death record indicated a TBI-related diagnosis. National Death Index record axis condition codes were used (both Part I and Part II of entity axis cause of death condition codes) (*3**)*. Data on TBI-related deaths were stratified by state and principal mechanism of injury (cause). Injuries were grouped first by intent (intentional, unintentional, or undetermined intent). Intentional injuries were further categorized as suicides or homicides. Unintentional injuries were categorized by mechanism of injury (including motor vehicle crash, fall, being struck by or against an object, or unspecified). Principal mechanism of injury was determined based on the CDC-recommended external cause of injury mortality matrix for ICD-10 codes (*4*) and reported as the average of the 3-year grouping. U.S. Census Bureau data were used to determine the percentage of each state’s population that lived in rural areas. Urban areas encompass at least 2,500 persons, at least 1,500 of whom live outside institutional group quarters; rural areas encompass all population, housing, and territory not included within an urban area^†^

Each state’s TBI-related death rates and corresponding 95% confidence interval (CI) were based on U.S. bridged-race population estimates of the resident population (*5*). U.S. Census population estimates for the year 2000 were used as the standard for age-adjusted rates by the direct method (*6*). The percentage difference between the overall TBI-related death rate for the United States and each state’s TBI-related death rate was calculated. Nonoverlapping CIs were used to analyze between-group differences for rates of TBI-related deaths. Because the use of nonoverlapping CIs is a conservative approach for determining statistically significant differences, a t-test was conducted if the 95% CI of two groups overlapped to determine whether there was a statistically significant difference. A Pearson correlation was calculated to determine the association between a state’s level of rurality and its TBI-related death rate. SAS (version 9.4; SAS Institute) was used for all analyses. This activity was reviewed by CDC and was conducted consistent with applicable federal law and CDC policy.^§^

During 2016–2018, a total of 181,227 TBI-related deaths occurred in the United States. The overall U.S. TBI-related death rate was 17.3 per 100,000 population per year (Table 1). The Northeast Census region had the lowest TBI-related death rate (12.8), followed by the West (16.8), and Midwest (18.1); the highest rate (19.2) was in the South.^¶^ The lowest state TBI-related death rate was in New Jersey (9.3); the highest rates were in Alaska (34.8), Wyoming (32.6), and Montana (29.5) (t-tests, p-value<0.05). State-specific TBI-related death rates were lower than the overall U.S. rate in 13 states and the District of Columbia (DC), higher in 35 states, and very close to the national average in one state (Pennsylvania). New Jersey’s TBI-related death rate was 46.2% lower than the overall U.S. rate, and Alaska’s rate was 101.2% higher.

**TABLE 1:** Estimated number,* age-adjusted rates,^†^ and percentage difference^§^ from overall U.S. rate of traumatic brain injury–related deaths,^¶^^,^** by state — United States, 2016–2018

Region^††^/State	No.	Rate (95% CI)	% Difference from overall U.S. rate
**U.S. total**	**181,227**	**17.3 (17.2–17.4)**	**NA**
Northeast	24,550	12.8 (12.6–12.9)	−26.0
Midwest	40,272	18.1 (17.9–18.3)	4.6
South	75,361	19.2 (19.1–19.4)	11.0
West	41,044	16.8 (16.6–17.0)	−2.9
Alabama	3,235	21.2 (20.5–22.0)	22.5
Alaska	752	34.8 (32.2–37.3)	101.2
Arizona	4,691	20.3 (19.7–20.9)	17.3
Arkansas	2,314	24.3 (23.3–25.3)	40.5
California	15,264	12.3 (12.1–12.4)	−28.9
Colorado	3,776	21.9 (21.2–22.6)	26.6
Connecticut	1,984	16.2 (15.4–16.9)	−6.4
Delaware	396	12.4 (11.1–13.6)	−28.3
District of Columbia	239	11.3 (9.8–12.7)	−34.7
Florida	13,647	18.4 (18.1–18.7)	6.4
Georgia	6,400	20.3 (19.8–20.8)	17.3
Hawaii	662	12.8 (11.8–13.8)	−26.0
Idaho	1,380	26.4 (25.0–27.8)	52.6
Illinois	5,395	13.0 (12.6–13.3)	−24.9
Indiana	3,835	18.3 (17.7–18.9)	5.8
Iowa	1,933	18.2 (17.3–19.0)	5.2
Kansas	1,985	21.3 (20.3–22.2)	23.1
Kentucky	3,232	23.2 (22.3–24.0)	34.1
Louisiana	3,109	21.6 (20.9–22.4)	24.9
Maine	1,069	22.5 (21.0–23.9)	30.1
Maryland	2,484	12.7 (12.2–13.2)	−26.6
Massachusetts	2,507	10.4 (10.0–10.9)	−39.9
Michigan	5,234	15.7 (15.3–16.2)	−9.2
Minnesota	2,754	14.8 (14.3–15.4)	−14.5
Mississippi	2,244	24.3 (23.2–25.3)	40.5
Missouri	4,818	24.8 (24.1–25.6)	43.4
Montana	993	29.5 (27.6–31.4)	70.5
Nebraska	1,290	20.9 (19.7–22.0)	20.8
Nevada	1,875	20.1 (19.2–21.1)	16.2
New Hampshire	856	18.6 (17.3–19.9)	7.5
New Jersey	2,752	9.3 (9.0–9.7)	−46.2
New Mexico	1,690	25.3 (24.1–26.6)	46.2
New York	6,714	10.1 (9.8–10.3)	−41.6
North Carolina	6,339	19.4 (18.9–19.9)	12.1
North Dakota	576	23.8 (21.7–25.8)	37.6
Ohio	7,787	20.5 (20.0–21.0)	18.5
Oklahoma	2,880	23.2 (22.4–24.1)	34.1
Oregon	2,919	21.3 (20.5–22.0)	23.1
Pennsylvania	7,688	17.4 (17.0–17.8)	0.6
Rhode Island	426	10.9 (9.8–12.0)	−37.0
South Carolina	4,310	27.3 (26.5–28.2)	57.8
South Dakota	690	25.0 (23.1–26.9)	44.5
Tennessee	4,392	20.6 (19.9–21.2)	19.1
Texas	13,546	16.2 (16.0–16.5)	−6.4
Utah	1,851	21.7 (20.7–22.7)	25.4
Vermont	554	26.2 (24.0–28.5)	51.4
Virginia	5,330	19.8 (19.3–20.4)	14.5
Washington	4,601	19.4 (18.9–20.0)	12.1
West Virginia	1,264	20.9 (19.7–22.1)	20.8
Wisconsin	3,975	20.4 (19.8–21.1)	17.9
Wyoming	590	32.6 (29.9–35.3)	88.4

**Abbreviations**: CI = confidence interval; ICD-10 = *International Classification of Diseases, Tenth Edition*; NA = not applicable.

* Deaths with missing age were excluded.

^†^ Per 100,000 population per year. Age-adjusted to the 2000 U.S. standard population. Adjustments made by 12 age groups: 0–4, 5–9, 10–14, 15–19, 20–24, 25–34, 35–44, 45–54, 55–64, 65–74, 74–84, and ≥85 years.

^§^ Differences in any two rates were considered statistically significant if their confidence intervals were not overlapping.

^¶^ Traumatic brain injuries are defined by the following ICD-10 diagnosis codes: S01, S02.0, S02.1, S02.3, S02.7-S02.9, S04.0, S06, S07.0, S07.1, S07.8, S07.9, S09.7-S09.9, T90.1, T90.2, T90.4, T90.5, T90.8, and T90.9.

** Record-Axis Condition codes were used (usually included both Part I and Part II of Entity-Axis Condition codes). Death estimates were obtained from CDC’s National Vital Statistics System.

†† https://www2.census.gov/geo/pdfs/maps-data/maps/reference/us_regdiv.pdf

States with higher percentages of rural residents had higher rates of TBI-related deaths (r = 0.65, p<0.0001) (Supplementary Figure, https://stacks.cdc.gov/view/cdc/110372). Overall, more than two in five (44.4%) TBI-related deaths were attributable to intentional causes (suicide and homicide). Suicide was the leading category or tied for the leading category of TBI-related deaths in 43 states (Table 2). In states where suicide was not the leading category of TBI-related deaths, unintentional falls were often responsible for the highest rate of TBI-related deaths (Table 3); unintentional falls were the second most common cause of TBI-related deaths in many states. Unintentional motor vehicle crashes and homicide were the third and fourth most common categories of TBI-related deaths, respectively, in the United States during 2016–2018.

**TABLE 2:** Estimated number* and age-adjusted rates^†^ of intentional traumatic brain injury–related deaths,^§^ by state and mechanism of injury — United States, 2016–2018^¶^

Region**/State	Intentional total		Suicide^††^		Homicide		Other^§§^	
	No.	Rate (95% CI)	No.	Rate (95% CI)	No.	Rate (95% CI)	No.	Rate (95% CI)
**U.S. total**	**80,479**	**8.0 (7.9–8.0)**	**62,985**	**7.1 (7.1–7.2)**	**17,494**	**1.8 (1.8–1.9)**	**2,571**	**0.3 (0.2–0.3)**
Northeast	8,510	4.8 (4.7–4.9)	6,463	4.1 (4.0–4.2)	2,047	1.2 (1.2–1.3)	448	0.2 (0.2–0.3)
Midwest	17,009	8.1 (8.0–8.2)	13,322	7.3 (7.1–7.4)	3,687	1.9 (1.8–1.9)	517	0.2 (0.2–0.3)
South	36,156	9.4 (9.3–9.5)	27,869	8.4 (8.3–8.5)	8,287	2.3 (2.2–2.3)	923	0.2 (0.2–0.3)
West	18,804	7.8 (7.7–7.9)	15,331	7.4 (7.2–7.5)	3,473	1.5 (1.4–1.5)	683	0.3 (0.3–0.3)
Alabama	1,807	12.1 (11.5–12.7)	1,328	10.1 (9.6–10.7)	479	3.4 (3.1–3.7)	49	0.3 (0.2–0.4)
Alaska	395	17.7 (15.9–19.5)	302	15.7 (13.9–17.5)	93	4.3 (3.4–5.2)	29	1.3 (0.8–1.7)
Arizona	2,513	11.2 (10.8–11.7)	2,137	11.0 (10.5–11.5)	376	1.8 (1.6–2.0)	120	0.6 (0.5–0.7)
Arkansas	1,174	12.7 (11.9–13.4)	906	11.2 (10.4–11.9)	268	3.1 (2.7–3.4)	34	0.4 (0.3–0.5)
California	6,149	5.0 (4.9–5.1)	4,466	4.2 (4.1–4.3)	1,683	1.4 (1.3–1.5)	217	0.2 (0.2–0.2)
Colorado	1,859	10.7 (10.2–11.2)	1,596	10.6 (10.1–11.2)	263	1.6 (1.4–1.8)	59	0.3 (0.2–0.4)
Connecticut	483	4.2 (3.8–4.6)	368	3.6 (3.2–4.0)	115	1.1 (0.9–1.3)	42	0.4 (0.3–0.5)
Delaware	198	6.7 (5.7–7.7)	150	5.7 (4.8–6.7)	48	1.8 (1.3–2.3)	—^¶¶^	—
District of Columbia	113	5.1 (4.1–6.0)	25	1.2 (0.7–1.6)	88	4.1 (3.2–5.0)	—	—
Florida	6,024	8.7 (8.5–8.9)	4,819	7.8 (7.6–8.1)	1,205	2.0 (1.9–2.1)	115	0.2 (0.1–0.2)
Georgia	3,113	9.8 (9.4–10.1)	2,380	8.6 (8.2–8.9)	733	2.4 (2.2–2.6)	62	0.2 (0.2–0.3)
Hawaii	136	3.0 (2.5–3.6)	95	2.4 (1.9–2.9)	41	1.0 (0.7–1.3)	16	—
Idaho	693	13.4 (12.4–14.5)	639	14.4 (13.3–15.6)	54	1.1 (0.8–1.4)	13	—
Illinois	2,137	5.4 (5.2–5.6)	1,469	4.2 (4.0–4.5)	668	1.8 (1.6–1.9)	77	0.2 (0.1–0.2)
Indiana	1,881	9.3 (8.9–9.7)	1,422	8.0 (7.6–8.5)	459	2.4 (2.2–2.6)	52	0.3 (0.2–0.3)
Iowa	672	6.9 (6.4–7.5)	586	7.0 (6.4–7.6)	86	0.9 (0.7–1.1)	19	—
Kansas	892	10.2 (9.5–10.9)	747	9.9 (9.2–10.6)	145	1.7 (1.4–2.0)	30	0.3 (0.2–0.4)
Kentucky	1,557	11.5 (10.9–12.0)	1,223	10.3 (9.7–10.9)	334	2.6 (2.3–2.9)	49	0.4 (0.3–0.5)
Louisiana	1,714	12.1 (11.5–12.7)	1,160	9.4 (8.8–10.0)	554	4.1 (3.7–4.4)	32	0.2 (0.1–0.3)
Maine	407	9.2 (8.2–10.1)	380	9.9 (8.8–10.9)	27	0.7 (0.4–0.9)	13	—
Maryland	990	5.3 (5.0–5.6)	654	3.9 (3.6–4.2)	336	1.9 (1.7–2.1)	40	0.2 (0.1–0.3)
Massachusetts	595	2.7 (2.5–2.9)	433	2.2 (2.0–2.5)	162	0.8 (0.7–0.9)	42	0.2 (0.1–0.3)
Michigan	2,360	7.5 (7.2–7.9)	1,914	7.0 (6.7–7.3)	446	1.5 (1.4–1.7)	68	0.2 (0.2–0.3)
Minnesota	1,045	6.1 (5.7–6.5)	931	6.3 (5.9–6.7)	114	0.7 (0.6–0.8)	47	0.3 (0.2–0.4)
Mississippi	1,053	11.6 (10.9–12.3)	753	9.4 (8.8–10.1)	300	3.5 (3.1–3.9)	41	0.5 (0.3–0.6)
Missouri	2,421	13.1 (12.5–13.6)	1,800	11.0 (10.5–11.6)	621	3.6 (3.3–3.9)	48	0.3 (0.2–0.3)
Montana	522	16.2 (14.8–17.7)	480	17.3 (15.7–18.9)	42	1.3 (0.9–1.8)	17	—
Nebraska	403	6.9 (6.2–7.6)	347	6.9 (6.1–7.6)	56	1.0 (0.7–1.3)	22	0.4 (0.2–0.5)
Nevada	1,122	12.1 (11.4–12.9)	934	11.6 (10.9–12.4)	188	2.2 (1.8–2.5)	48	0.5 (0.4–0.6)
New Hampshire	385	8.9 (8.0–9.8)	358	9.6 (8.5–10.6)	27	0.7 (0.4–1.0)	—	—
New Jersey	920	3.4 (3.1–3.6)	538	2.2 (2.0–2.4)	382	1.5 (1.3–1.6)	46	0.2 (0.1–0.2)
New Mexico	888	13.6 (12.7–14.5)	694	12.1 (11.2–13.1)	194	3.2 (2.7–3.6)	50	0.8 (0.6–1.0)
New York	2,100	3.4 (3.3–3.6)	1,461	2.7 (2.5–2.8)	639	1.1 (1.0–1.2)	190	0.3 (0.3–0.3)
North Carolina	2,795	8.8 (8.4–9.1)	2,132	7.7 (7.3–8.0)	663	2.2 (2.0–2.3)	64	0.2 (0.2–0.3)
North Dakota	236	10.2 (8.9–11.6)	214	10.8 (9.3–12.3)	22	0.9 (0.5–1.3)	—	—
Ohio	3,300	9.2 (8.9–9.5)	2,490	7.9 (7.6–8.2)	810	2.4 (2.2–2.6)	93	0.3 (0.2–0.3)
Oklahoma	1,500	12.5 (11.8–13.1)	1,233	11.9 (11.2–12.5)	267	2.3 (2.0–2.6)	36	0.3 (0.2–0.4)
Oregon	1,327	10.0 (9.5–10.6)	1,188	10.4 (9.8–11.0)	139	1.1 (0.9–1.3)	24	0.2 (0.1–0.3)
Pennsylvania	3,316	8.2 (7.9–8.5)	2,659	7.5 (7.2–7.8)	657	1.8 (1.7–1.9)	82	0.2 (0.2–0.2)
Rhode Island	98	2.8 (2.3–3.4)	80	2.7 (2.1–3.3)	18	0.5 (0.3–0.8)	15	—
South Carolina	1,841	11.9 (11.3–12.4)	1,333	9.8 (9.2–10.3)	508	3.5 (3.2–3.8)	35	0.2 (0.1–0.3)
South Dakota	270	10.6 (9.3–11.8)	226	10.2 (8.9–11.6)	44	1.8 (1.2–2.3)	—	—
Tennessee	2,192	10.6 (10.1–11.0)	1,753	9.7 (9.2–10.2)	439	2.2 (2.0–2.5)	78	0.4 (0.3–0.5)
Texas	7,038	8.3 (8.1–8.5)	5,589	7.7 (7.5–7.9)	1,449	1.7 (1.6–1.8)	202	0.2 (0.2–0.3)
Utah	967	11.0 (10.3–11.7)	878	11.6 (10.9–12.4)	89	1.0 (0.8–1.2)	34	0.4 (0.2–0.5)
Vermont	206	10.5 (9.0–12.0)	186	10.9 (9.2–12.5)	20	1.2 (0.6–1.7)	—	—
Virginia	2,330	8.8 (8.4–9.2)	1,828	7.9 (7.6–8.3)	502	2.0 (1.8–2.2)	58	0.2 (0.2–0.3)
Washington	1,944	8.3 (7.9–8.7)	1,658	8.2 (7.8–8.6)	286	1.3 (1.1–1.4)	54	0.2 (0.2–0.3)
West Virginia	717	12.6 (11.6–13.5)	603	12.1 (11.1–13.1)	114	2.2 (1.8–2.6)	22	0.4 (0.2–0.5)
Wisconsin	1,392	7.7 (7.3–8.2)	1,176	7.5 (7.0–7.9)	216	1.3 (1.2–1.5)	50	0.3 (0.2–0.4)
Wyoming	289	16.4 (14.5–18.4)	264	17.4 (15.2–19.5)	25	1.5 (0.9–2.1)	—	—

**Abbreviation**: CI = confidence interval.

* Deaths with missing age were excluded.

^†^ Per 100,000 population per year. Age-adjusted to the 2000 U.S. standard population. Adjustments made by 12 age groups: 0–4, 5–9, 10–14, 15–19, 20–24, 25–34, 35–44, 45–54, 55–64, 65–74, 74–84, and ≥85 years.

^§^ Record-Axis Condition codes were used (usually included both Part I and Part II of Entity-Axis Condition codes). Death estimates obtained from CDC’s National Vital Statistics System.

^¶^ Differences in any two rates were considered statistically significant if their confidence intervals were not overlapping.

** https://www2.census.gov/geo/pdfs/maps-data/maps/reference/us_regdiv.pdf

^††^ Suicides reported in persons aged <10 years were excluded; whether children aged <10 years are able to form suicidal intent is unclear.

^§§^ Other indicates that no intent or mechanism was specified in the record and includes traumatic brain injuries in which the intent was not determined, falls of undetermined intent, and those because of legal intervention or war.

^¶¶^ Dashes indicate values suppressed for counts ≤10 and rates based on <20 count.

**TABLE 3:** Estimated number* and age-adjusted rates^†^ of unintentional traumatic brain injury–related deaths,^§^ by state and mechanism of injury — United States, 2016–2018^¶^

Region**/State	Total		Motor vehicle crashes		Falls^††^		Struck by or against an object		Other^§§^	
	No.	Rate (95% CI)	No.	Rate (95% CI)	No.	Rate (95% CI)	No.	Rate (95% CI)	No.	Rate (95% CI)
**U.S. total**	**98,177**	**9.1 (9.0–9.1)**	**33,152**	**3.3 (3.3–3.4)**	**51,903**	**4.5 (4.5–4.5)**	**992**	**0.1 (0.1–0.1)**	**12,130**	**1.1 (1.1–1.2)**
Northeast	15,592	7.7 (7.6–7.9)	4,411	2.5 (2.5–2.6)	9,267	4.2 (4.1–4.3)	156	0.1 (0.1–0.1)	1,758	0.9 (0.9–0.9)
Midwest	22,746	9.8 (9.6–9.9)	7,078	3.4 (3.3–3.5)	12,449	4.9 (4.8–5.0)	253	0.1 (0.1–0.1)	2,966	1.3 (1.3–1.4)
South	38,282	9.5 (9.5–9.6)	14,723	3.9 (3.9–4.0)	18,427	4.3 (4.3–4.4)	380	0.1 (0.1–0.1)	4,752	1.2 (1.2–1.2)
West	21,557	8.7 (8.6–8.8)	6,940	2.9 (2.9–3.0)	11,760	4.6 (4.5–4.7)	203	0.1 (0.1–0.1)	2,654	1.1 (1.0–1.1)
Alabama	1,379	8.8 (8.3–9.2)	615	4.3 (3.9–4.6)	435	2.5 (2.2–2.7)	13	—^¶¶^	316	1.9 (1.7–2.2)
Alaska	328	15.8 (14.0–17.6)	148	6.6 (5.5–7.7)	105	5.8 (4.6–6.9)	—^¶¶^	—	72	3.3 (2.5–4.1)
Arizona	2,058	8.5 (8.1–8.9)	575	2.7 (2.5–2.9)	1,254	4.8 (4.5–5.1)	—	—	221	1.0 (0.9–1.1)
Arkansas	1,106	11.3 (10.6–12.0)	491	5.5 (5.0–6.0)	461	4.2 (3.8–4.6)	20	0.2 (0.1–0.3)	134	1.4 (1.2–1.6)
California	8,898	7.1 (6.9–7.2)	2,812	2.3 (2.2–2.4)	4,832	3.8 (3.7–3.9)	86	0.1 (0.1–0.1)	1,168	0.9 (0.9–1.0)
Colorado	1,858	10.8 (10.3–11.3)	543	3.1 (2.9–3.4)	1,049	6.2 (5.8–6.6)	12	—	254	1.4 (1.3–1.6)
Connecticut	1,459	11.6 (11.0–12.2)	623	5.7 (5.2–6.1)	632	4.4 (4.0–4.7)	12	—	192	1.4 (1.2–1.6)
Delaware	194	5.5 (4.7–6.3)	37	1.2 (0.8–1.7)	131	3.5 (2.9–4.1)	—	—	26	0.7 (0.5–1.0)
District of Columbia	124	6.1 (5.0–7.2)	23	1.0 (0.6–1.5)	81	4.0 (3.1–4.9)	—	—	20	1.0 (0.5–1.4)
Florida	7,508	9.5 (9.3–9.7)	2,613	4.1 (4.0–4.3)	4,128	4.3 (4.2–4.5)	31	0.0 (0.0–0.1)	736	1.0 (0.9–1.1)
Georgia	3,225	10.4 (10.0–10.7)	1,491	4.7 (4.5–4.9)	1,299	4.3 (4.1–4.6)	36	0.1 (0.1–0.1)	399	1.3 (1.1–1.4)
Hawaii	510	9.4 (8.5–10.3)	132	3.0 (2.5–3.6)	327	5.3 (4.7–5.9)	—	—	49	1.0 (0.7–1.3)
Idaho	674	12.7 (11.7–13.6)	299	5.9 (5.2–6.6)	297	5.3 (4.7–5.9)	—	—	68	1.3 (1.0–1.6)
Illinois	3,181	7.4 (7.1–7.6)	908	2.3 (2.2–2.5)	1,895	4.2 (4.0–4.4)	30	0.1 (0.0–0.1)	348	0.8 (0.7–0.9)
Indiana	1,902	8.7 (8.3–9.1)	689	3.4 (3.2–3.7)	904	3.9 (3.6–4.1)	29	0.1 (0.1–0.2)	280	1.3 (1.1–1.4)
Iowa	1,242	11.0 (10.4–11.7)	330	3.4 (3.1–3.8)	762	6.1 (5.7–6.6)	15	—	135	1.3 (1.1–1.5)
Kansas	1,063	10.7 (10.1–11.4)	314	3.6 (3.1–4.0)	599	5.6 (5.1–6.0)	—	—	140	1.5 (1.2–1.7)
Kentucky	1,626	11.3 (10.8–11.9)	711	5.3 (4.9–5.7)	625	4.1 (3.7–4.4)	28	0.2 (0.1–0.3)	262	1.8 (1.6–2.1)
Louisiana	1,363	9.3 (8.8–9.8)	556	4.0 (3.7–4.3)	590	3.8 (3.5–4.1)	14	—	203	1.4 (1.2–1.6)
Maine	649	13.0 (12.0–14.1)	206	5.2 (4.5–6.0)	385	6.4 (5.8–7.1)	—	—	52	1.2 (0.9–1.6)
Maryland	1,454	7.2 (6.8–7.6)	265	1.4 (1.3–1.6)	1,038	5.0 (4.7–5.3)	23	0.1 (0.1–0.2)	128	0.6 (0.5–0.8)
Massachusetts	1,870	7.5 (7.2–7.9)	421	1.9 (1.7–2.1)	1,270	4.8 (4.6–5.1)	10	—	169	0.7 (0.6–0.8)
Michigan	2,806	8.0 (7.7–8.3)	631	2.1 (1.9–2.2)	1,529	4.1 (3.9–4.3)	28	0.1 (0.1–0.1)	618	1.8 (1.6–1.9)
Minnesota	1,662	8.4 (8.0–8.9)	234	1.4 (1.2–1.6)	1,235	6.0 (5.7–6.3)	11	—	182	1.0 (0.8–1.1)
Mississippi	1,150	12.2 (11.5–12.9)	532	6.0 (5.5–6.5)	464	4.5 (4.1–5.0)	—	—	146	1.6 (1.3–1.9)
Missouri	2,349	11.5 (11.0–12.0)	1,009	5.4 (5.1–5.8)	1,017	4.5 (4.2–4.7)	32	0.2 (0.1–0.2)	291	1.5 (1.3–1.6)
Montana	454	12.8 (11.6–14.0)	187	5.9 (5.0–6.7)	199	5.0 (4.3–5.7)	—	—	65	1.9 (1.4–2.4)
Nebraska	865	13.6 (12.6–14.5)	355	6.2 (5.5–6.8)	385	5.4 (4.9–6.0)	—	—	117	1.8 (1.5–2.2)
Nevada	705	7.5 (7.0–8.1)	147	1.6 (1.3–1.9)	465	4.9 (4.5–5.4)	—	—	89	0.9 (0.7–1.1)
New Hampshire	462	9.5 (8.6–10.3)	149	3.4 (2.8–4.0)	262	5.0 (4.4–5.6)	—	—	46	0.9 (0.7–1.2)
New Jersey	1,786	5.8 (5.5–6.1)	525	1.9 (1.8–2.1)	993	3.0 (2.8–3.2)	—	—	259	0.8 (0.7–0.9)
New Mexico	752	10.9 (10.1–11.7)	224	3.7 (3.2–4.2)	429	5.7 (5.2–6.3)	—	—	92	1.4 (1.1–1.6)
New York	4,424	6.4 (6.2–6.6)	1,239	2.0 (1.9–2.1)	2,608	3.5 (3.4–3.7)	50	0.1 (0.1–0.1)	527	0.8 (0.7–0.9)
North Carolina	3,480	10.5 (10.1–10.8)	1,307	4.2 (4.0–4.5)	1,705	4.8 (4.6–5.1)	37	0.1 (0.1–0.1)	431	1.3 (1.2–1.4)
North Dakota	333	13.3 (11.8–14.8)	162	6.9 (5.8–8.0)	122	4.2 (3.4–4.9)	—	—	44	2.0 (1.4–2.6)
Ohio	4,394	11.0 (10.7–11.4)	1,581	4.5 (4.3–4.7)	2,236	5.1 (4.8–5.3)	50	0.1 (0.1–0.2)	527	1.4 (1.2–1.5)
Oklahoma	1,344	10.4 (9.9–11.0)	318	2.6 (2.3–2.9)	842	6.3 (5.8–6.7)	—	—	175	1.4 (1.2–1.7)
Oregon	1,568	11.0 (10.5–11.6)	564	4.4 (4.0–4.7)	835	5.4 (5.1–5.8)	28	0.2 (0.1–0.3)	141	1.0 (0.8–1.2)
Pennsylvania	4,290	9.0 (8.7–9.2)	1,080	2.8 (2.6–2.9)	2,701	5.0 (4.8–5.2)	57	0.1 (0.1–0.2)	452	1.0 (0.9–1.1)
Rhode Island	313	7.7 (6.8–8.6)	46	1.4 (1.0–1.8)	236	5.4 (4.7–6.1)	—	—	30	0.9 (0.5–1.2)
South Carolina	2,434	15.2 (14.6–15.8)	1,352	8.9 (8.5–9.4)	820	4.7 (4.3–5.0)	20	0.1 (0.1–0.2)	242	1.5 (1.3–1.7)
South Dakota	416	14.3 (12.9–15.7)	139	5.3 (4.4–6.2)	225	7.2 (6.2–8.1)	—	—	47	1.7 (1.2–2.2)
Tennessee	2,122	9.6 (9.2–10.0)	623	3.1 (2.8–3.3)	1,102	4.7 (4.4–5.0)	28	0.1 (0.1–0.2)	369	1.7 (1.5–1.8)
Texas	6,306	7.7 (7.5–7.9)	2,367	2.8 (2.7–2.9)	3,096	3.9 (3.8–4.1)	56	0.1 (0.0–0.1)	787	0.9 (0.9–1.0)
Utah	850	10.4 (9.6–11.1)	346	3.8 (3.4–4.2)	391	5.2 (4.7–5.7)	—	—	104	1.2 (1.0–1.5)
Vermont	339	15.2 (13.5–16.9)	122	6.4 (5.2–7.6)	180	7.2 (6.1–8.3)	—	—	31	1.4 (0.9–1.9)
Virginia	2,942	10.8 (10.4–11.2)	1,283	4.9 (4.7–5.2)	1,310	4.6 (4.4–4.9)	42	0.2 (0.1–0.2)	307	1.1 (1.0–1.2)
Washington	2,603	10.9 (10.5–11.3)	830	3.7 (3.4–3.9)	1,446	5.9 (5.6–6.2)	27	0.1 (0.1–0.2)	300	1.2 (1.1–1.4)
West Virginia	525	7.9 (7.2–8.7)	139	2.5 (2.0–2.9)	300	4.0 (3.6–4.5)	15	—	71	1.2 (0.9–1.5)
Wisconsin	2,533	12.4 (11.9–12.9)	726	4.0 (3.7–4.3)	1,540	7.0 (6.6–7.3)	30	0.1 (0.1–0.2)	237	1.3 (1.1–1.4)
Wyoming	299	16.1 (14.2–18.0)	133	7.9 (6.5–9.2)	131	6.5 (5.4–7.7)	—	—	31	1.5 (1.0–2.1)

**Abbreviation**: CI = confidence interval.

* Deaths with missing age were excluded.

^†^ Per 100,000 population per year. Age-adjusted to the 2000 U.S. standard population. Adjustments made by 12 age groups: 0–4, 5–9, 10–14, 15–19, 20–24, 25–34, 35–44, 45–54, 55–64, 65–74, 74–84, and ≥85 years.

^§^ Record-Axis Condition codes were used (usually included both Part I and Part II of Entity-Axis Condition codes). Death estimates obtained from CDC’s National Vital Statistics System.

^¶^ Differences in any two rates were considered statistically significant if their confidence intervals were not overlapping.

** https://www2.census.gov/geo/pdfs/maps-data/maps/reference/us_regdiv.pdf

^††^ Excluded falls of undetermined intent.

^§§^ External cause of injury codes specify that the injury was unintentional but do not specify the actual mechanism of injury.

^¶¶^ Dashes indicate values suppressed for counts ≤10 and rates based on <20 count.

## Discussion

During 2016–2018, approximately 180,000 TBI-related deaths occurred in the United States. Rates of TBI-related deaths rates differed considerably by state during this period. The four states with the lowest rates were in the Northeast; the rates in these states were at least 35% lower than the overall U.S. rate. The three states with the highest rates were in the West and were at least 70% higher than the overall U.S. rate. This pattern occurred both for intentional and unintentional causes of TBI-related deaths. 

Level of rurality might play a role in the incidence of TBI-related deaths in states with higher rates. Residents in rural areas experience a higher incidence of TBI (*2*) and might face barriers to accessing emergency medical care (including Level I trauma centers) (*7*) and specialized TBI care (*8*). Disparate TBI-related death rates might also result from risk differences in mechanisms of injury and implementation of state injury prevention policies. Although seat belt use is associated with a 50% reduction in deaths from a motor vehicle crash, legislation and enforcement vary among states (*9*). CDC’s Motor Vehicle Prioritizing Interventions and Cost Calculator for States (MVPICCS) tool identifies effective motor vehicle injury prevention strategies that states can implement.**

Approximately 40% of TBI-related deaths examined were categorized as intentional injuries (i.e., homicides or suicides). Suicide was responsible for the highest number and the highest rate of TBI-related deaths for most states. The rates of TBI-related deaths due to homicide in some southern jurisdictions (e.g., Louisiana, DC, and South Carolina) were nearly double those in many states in the Northeast. A previous report found that nearly all TBI-related deaths from suicide had firearm injury as the underlying mechanism of injury (*1*). These patterns correspond with regional analyses indicating that firearm-related homicide rates are highest in the South and firearm-related suicide rates are highest in the South and West (*10*). State and community implementation of a multipronged approach, including evidence-based violence prevention strategies, such as those identified in CDC’s technical packages on violence and suicide prevention,^††^ could be considered to reduce suicides and homicides, including those that are TBI-related.

Unintentional falls were the second most common cause of TBI-related deaths in many states. Evidence-based, cost-effective prevention efforts could be implemented to decrease fall-related injuries and deaths, most commonly experienced by older adults. CDC’s Stopping Elderly Accidents, Deaths and Injuries (STEADI) initiative includes resources and tools for health care providers that are designed to improve identification of patients at risk for a fall and support implementation of effective strategies to treat or reduce the risk for fall-related injuries, including TBI.^§§^

The findings in this report are subject to at least three limitations. First, incomplete reporting or misclassification of cause of death (i.e., deaths from other causes or deaths indirectly caused by TBI) on death certificates might bias estimates of TBI-related deaths. For example, NVSS data might not include all TBI-related deaths, especially if a TBI was not explicitly documented in death records. Second, data were combined across multiple years; therefore, a trend analysis was not possible; different patterns have emerged over time. Finally, data presented in this report are from the years 2016–2018, and might not reflect more recent differences. The COVID-19 pandemic, in particular, might have had an impact on more recent estimates.

Expanding evidence-based prevention strategies that address TBI-related deaths is warranted, especially among states with high rates attributable to suicide, unintentional falls, and motor vehicle crashes. States can employ a number of evidence-based strategies (such as those identified in CDC’s technical packages on violence and suicide prevention) to reduce the leading causes of TBI-related deaths, including those for suicide,^¶¶^ unintentional falls,*** and motor vehicle crashes.^†††^

**Summary**

**What is already known about this topic?**

Traumatic brain injuries (TBIs) have contributed to approximately one million U.S. deaths during the last 2 decades. Rates of TBIs vary by state.

**What is added by this report?**

During 2016–2018, states in the Northeast had the lowest TBI–related death rates (12.8 per 100,000), whereas rates were highest in the South (19.2). Suicide and unintentional falls contributed the highest number of TBI-related deaths in most states. States with a large proportion of residents living in rural areas had higher TBI-related death rates.

**What are the implications for public health practice?**

Expanding evidence-based prevention strategies that address TBI-related deaths is warranted, especially among states with high rates due to suicide, unintentional falls, and motor vehicle crashes.

## Footnotes

* Traumatic brain injuries are defined by the following ICD-10 diagnosis codes: S01, S02.0, S02.1, S02.3, S02.7-S02.9, S04.0, S06, S07.0, S07.1, S07.8, S07.9, S09.7-S09.9, T90.1, T90.2, T90.4, T90.5, T90.8, and T90.9. https://www.cdc.gov/injury/wisqars/mapping_help/injury_type.html

†
https://www.census.gov/programs-surveys/geography/guidance/geo-areas/urban-rural/2010-urban-rural.html

§ 45 C.F.R. part 46, 21 C.F.R. part 56; 42 U.S.C. Sect. 241(d); 5 U.S.C. Sect. 552a; 44 U.S.C. Sect. 3501 et seq.

¶
https://www2.census.gov/geo/pdfs/maps-data/maps/reference/us_regdiv.pdf

**
https://www.cdc.gov/transportationsafety/calculator/index.html

††
https://www.cdc.gov/violenceprevention/communicationresources/pub/technical-packages.html

§§
www.cdc.gov/STEADI

¶¶
 https://www.cdc.gov/suicide/index.html

***
https://www.cdc.gov/transportationsafety/index.html

†††
https://www.cdc.gov/homeandrecreationalsafety/falls/adultfalls.html
